# Changing the paradigm of cervical cancer prevention through introduction of HPV-testing: evaluation of the implementation process of the Jujuy Demonstration Project in Argentina

**DOI:** 10.3332/ecancer.2021.1199

**Published:** 2021-03-04

**Authors:** Silvina Arrossi, Melisa Paolino, Rosa Laudi, Laura Thouyaret

**Affiliations:** 1 Centro de Estudios de Estado y Sociedad/Consejo Nacional de Investigaciones Científicas y Técnicas, Sánchez de Bustamante 27, Buenos Aires 1193, Argentina; 2 Hospital Ramos Mejía, Urquiza 609, Buenos Aires 1221, Argentina; 3 Programa Nacional de Prevención de Cáncer Cervicouterino/Instituto Nacional del Cáncer (Argentina), Julio A, Roca 781, Buenos Aires 1067, Argentina

**Keywords:** cervical cancer, human papillomavirus DNA test, implementation, public health, Argentina

## Abstract

**Introduction:**

The Jujuy Demonstration Project (JDP) was a project carried out over the course of 4 years (2011–2014) to develop, implement and evaluate the programmatic components of a Human Papilloma Virus (HPV)-based screening programme in Argentina. The aim of this paper is to present a qualitative evaluation of the context and implementation process of the JDP.

**Methods:**

We used an adaptation of the Health System Framework (HSF), which includes interconnected contextual factors that are considered key drivers for successful health interventions. We reviewed secondary documents, which included scientific reports, norms and regulations, information sheets, power point presentations and manuals and recommendations published by the National Programme for Cervical Cancer Prevention. We also carried out semi-structured interviews with key informants to explore their views about technology acceptability.

**Results:**

Key components of the JDP implementation process were: a high level of political support and consensus among stakeholders, the demonstrated effectiveness of the technology and its acceptability by health authorities and providers, the funding of tests and diagnosis/treatment services, the implementation of an information system for monitoring and evaluation and the reorganisation of the network of screening, diagnosis and treatment services.

**Conclusion:**

This analysis examines the policy context in which the JDP was implemented and the system components that were key for the demonstrated effectiveness of the strategy. Such analyses provide useful insights into core components of HPV testing implementation that are needed to guarantee its potential effectiveness to reduce cervical cancer incidence and mortality.

## Introduction

Cervical cancer is almost completely preventable with existing knowledge and technology. Although the disease may be controlled with cytology-based screening [1] performed in an organised programme [2], such programmes have been difficult to implement in the health systems of low- and middle-income countries [3–4]. In the last decades, Human Papilloma Virus (HPV) testing was developed as an alternative screening method. The HPV test is highly sensitive and effective in preventing cervical cancer incidence and mortality [5]. In addition, HPV testing allows for self-collection of samples, which has proved effective in increasing screening [6, 7]. All of these features have made HPV testing the preferred tool for cervical cancer screening. In combination with HPV vaccination, HPV testing appears as a technology that could make a major contribution to the elimination of cervical cancer [8].

In programmatic contexts, the success of HPV testing will depend on the same factors that have affected the effectiveness of cytology-based screening [9]: the existence of a national policy; the adherence of the medical community to recommendations and protocols; the availability of economic/technical/human resources; the development of information/monitoring systems; high coverage/ treatment levels and a good quality diagnosis and treatment network. Thus, in low-/middle-income settings, for HPV testing to be effective, it has to be introduced in the context of a major system change, i.e. involving changes at all levels of the health system, the incorporation of stakeholders and the reorganisation of programmes and services [10]. Otherwise, HPV-based screening programmes will very likely not be able to reproduce the level of effectiveness obtained in controlled trials.

In Argentina, HPV testing as primary screening was introduced in 2012 through the Jujuy Demonstration Project (JDP) [11], a 4-year population-based study to evaluate large-scale programmatic introduction of HPV testing. Jujuy, the province chosen for the demonstration project, had high cervical cancer mortality (11.8 per 100,000 in 2008–10) [11]. Additionally, a situational analysis done in 2007 [12] showed that in Jujuy, cervical screening coverage was low, information systems were unreliable, information on follow-up and treatment was missing and providers showed low adherence to programmatic norms and recommendations. Cytology results were read in six laboratories, which processed in total around 22,000 annual samples without quality controls. To give an indication of the scale of the changes implemented through the JDP, starting 1 January 2012, all 300 provincial health care centres stopped doing cytology-based screening and began to offer HPV testing. Within 3 years of its implementation, over 49,000 women had been tested for HPV, 10% through self-collection. Compared with what had been achieved during the preceding cytology-based period, HPV testing more than doubled detection of cervical intraepithelial neoplasia grade 2 or worse (CIN2+) lesions and, in total, more than 500 women were treated for pre-neoplastic disease [11]. This increased detection was achieved in the context of programme restructuring that included laboratory and referral network reorganisation, the use of sample self-collection to increase coverage and the development of mechanisms to assure provider adherence to guidelines. Findings from the JDP showed that effective screening with HPV testing in real-world programmes of middle-income settings is feasible; based on its results, HPV-testing was scaled-up nationally, and by the year 2019, ten provinces were using the HPV test as primary screening.

In a complex intervention like the JDP, outcomes are often only partially related to the intervention itself; contextual factors/processes play a key and often dominant role and therefore it is vital to analyse how they influence implementation. In this paper, we evaluate the context and implementation process of the JDP. Research on systems is key to provide policy makers and practitioners with robust and relevant evidence that takes adequate account of the real-world circumstances in which policies are made and interventions are implemented [13]. Combining this health system analysis with the data on effectiveness that has been already published will allow for a better understanding of what components are key in order to reproduce in other settings the programmatic outcomes of the JDP.

## Methods

### Setting

Argentina is a democratic, federal country made up of 24 provinces, each of which is an autonomous entity responsible for the organisation, management and financing of the provincial health system. Among other activities, the National Ministry of Health provides a regulatory framework for health care provision, as well as training and financing for specific programmes through nationally and internationally-funded programmes. Provincial health ministries can choose whether or not to adhere to the proposed national health programmes or activities; their adherence mainly depends on formal agreements in which responsibilities and funding are negotiated. The province of Jujuy is located in Northwest Argentina and has around 673,000 inhabitants; 85% of its population lives in urban areas and 32% of its population is poor. The Jujuy health system includes a main hospital (Pablo Soria Hospital) and 300 primary health care (PHC) centres. The PHC System employs around 700 full-time community health workers (CHWs) who visit approximately 110,000 households twice yearly for health-related tasks such as immunisation and maternal and child health promotion.

**Figure 1.**
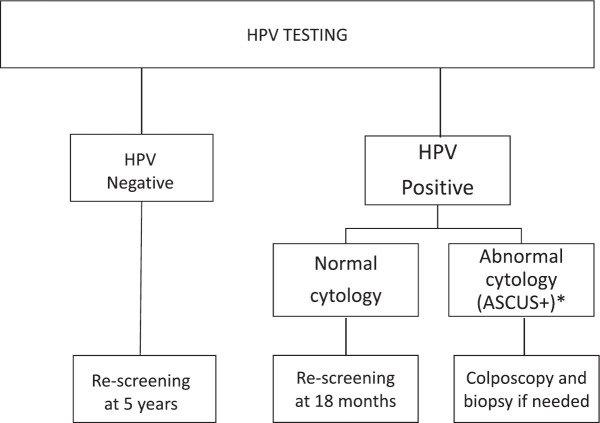
Cervical cancer screening algorithm for women aged 30+ with HPV testing. *ASCUS+ includes: Atypical squamous cells of undetermined significance (ASCUS), low-grade squamous intraepithelial lesion (LSIL); Atypical squamous cells cannot exclude HSIL (ASC-H); High grade squamous intraepithelial lesion (HSIL); Atypical glandular cells (AGC) and adenocarcinoma *in situ*.

The JDP introduced HPV testing as the primary screening strategy for women aged 30 and over receiving care in the public health sector. Before the introduction of HPV testing, cytology was the standard of care for screening. The protocols for HPV testing have been described elsewhere [14] and are shown in Figure 1. In 2014, HPV sample self-collection was introduced as a programmatic strategy mainly for under-screened women: those without screening in the last 5 years and with public health insurance [15]. For HPV self-collected tests, women with a positive result were to attend health centres to undergo cytology. The final analysis of the impact of the JDP has been published elsewhere [11]; key outcomes of the project are summarised in Table 1.

### Conceptual framework

A qualitative study was carried out to evaluate the implementation process of the JDP. For this we used an adaptation of the Health System Framework (HSF) [16–18], which includes interconnected contextual factors that are considered key drivers for successful health interventions [19]. The HSF integrates six building blocks (service delivery, health workforce, information, technology, funding and stewardship) that influence system performance; they were re-configured to incorporate an organisational dimension, which is a key factor for cervical cancer prevention programme effectiveness [20]. Thus, this adaptation presents four blocks (dimensions) instead of the original six, with specific sub-dimensions (Table 2). This adaptation of the HSF has already been used for analysis of the scaling-up of HPV self-collection in Argentina [15], as follows:

1. **Stewardship** refers to the policy environment in which the implementation of a health strategy is made possible [21]; sub-dimensions are: *Policy support*, *regulation*, *policy guidance* and *accountability*.
2. **Organisational capacity** refers to the capacity of the health system to implement the intervention in all stages of the screening/diagnoses/treatment continuum; sub-dimensions are: *Service delivery and health workforce* and* Information.*
3. **Health care financing** implies assuring funds for health in ways that guarantee people can use needed services [16]; its sub-dimension is: *Availability of sustainable funding for the strategy.*
4. **Technology/strategy** refers to access to essential medical products/vaccines/technologies and their scientifically sound and cost-effective use [16]; sub-dimensions are: *Acceptability* and* effectiveness and consensus about the value of the technology.*

### Data collection

Data sources are shown in Table 2. Review of secondary documents included scientific reports, information sheets, power point presentations, norms and regulations, manuals and recommendations published by the National Programme for Cervical Cancer Prevention (NPCCP) and notes taken during meetings and management roundtables. Also, during October–November 2013, we carried out ten semi-structured interviews with key informants to explore their views about technology acceptability: Minister of Health (1), Primary Health Care Director (1); Director of Maternity Health (1); Chief of Laboratory (1), Provincial Programme Coordinator (1) and health professionals (5). Informed consent was sought prior to each interview.

### Data analysis

Secondary documents and notes taken during participation of the implementation process were reviewed. Data from semi-structured interviews were transcribed. Data coding followed predetermined themes based on HSF dimensions and sub-dimensions. To ensure coding reliability, two researchers independently coded and performed consistency checks. After coding, thematic and content analysis was conducted [22]. Qualitative data were validated through triangulation of data sources.

Method details and interview results are presented following the Standards for Reporting Implementation Studies: The StaRI checklist for completion [23].

### Ethical statement

This protocol has been approved by the Centro de Educación Médica e Investigaciones (CEMIC) Institutional Review Board (Protocol number 1186). A written informed consent was obtained from all participants in the study.

## Results

**Figure 2.**
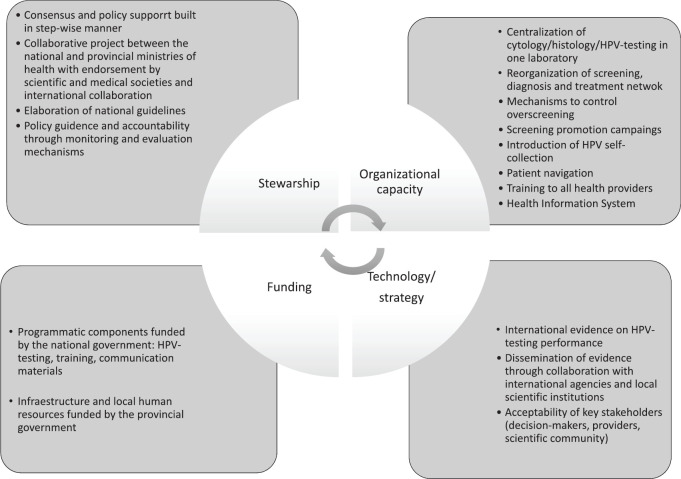
HSF for analysis of cervical cancer prevention.

Main results regarding the four components of the HSF are presented in Figure 2 and described in the following sections.

### Stewardship

#### Consensus building and political support

Consensus building and political support were key activities during the planning phase, carried out in a stepwise manner. Four phases were identified in the process: 1) developing a legitimised proposal to introduce HPV testing; 2) obtaining support from the National Ministry of Health; 3) obtaining support from the provincial health minister of Jujuy and agreement on resource provision and health system reorganisation; 4) involvement of second-level health authorities in Jujuy and the heads of the different health services.

First, introduction of HPV testing was approved by consensus by programme managers and health providers of the 24 Argentinean provinces [24] during the NPCCP’s annual scientific seminar in 2010 that also gathered international scientific leaders. Scientific evidence about HPV test performance was presented and discussed in this meeting and was the basis for the decision to introduce the test. Thus, the NPCCP got a mandate from meeting participants to implement a demonstration project to introduce HPV testing.

Secondly, the proposal was presented to the highest national health authorities (Under-secretary of Health and authorities of the National Cancer Institute), who saw the JDP as an opportunity to show action in women’s health, as well as to increase the visibility of the recently created National Cancer Institute. The National Ministry of Health approved the project and committed needed resources.

The introduction of HPV vaccination that same year (2011) was also a driver of HPV testing implementation. HPV testing was presented as part of a comprehensive national strategy to prevent cervical cancer that consisted of three components: HPV testing, HPV vaccination and a National and Regional Reference Laboratory for HPV (National Institute of Infectious Diseases – ANLIS Malbrán). A key message sent to health providers and managers was that in the context of HPV vaccination, cytology sensitivity was expected to decrease and therefore HPV testing was the recommended screening test.

Once national support was guaranteed, the project was presented to the Ministry of Health of Jujuy. A driving idea present in these initial discussions was the possibility of introducing a novel technology that would allow the provincial authorities to show actions and results towards reducing the province’s high burden of cervical cancer. An agreement was signed between the national and provincial Ministries of Health, which included as key elements the provision of tests and part of the treatment equipment by the national government, as well as communication materials and funding of specific human resources (data entry clerks and patient navigators). The provincial Ministry of Health agreed to reorganise health services as needed (i.e. laboratory centralisation) and commit resources to achieve the project goals (i.e. establishing cervical cancer screening as a priority activity for CHWs).

Finally, specific activities were carried out to involve second-level health authorities in Jujuy and the heads of health services. Several meetings were carried out to explain the project and to discuss goals, specific tasks and responsibilities. These were then formalised in an agreement signed between the national and provincial Ministries of Health [25].

#### Partnerships for collaboration

The JDP was established as a collaborative project between the national and provincial Ministries of Health, with the active participation of key health departments (i.e. PHC) and stakeholders at a very early stage of the design and planning process.

Endorsement from the scientific medical community was secured through a Scientific Advisory Committee (SAC), with the participation of the main national and provincial scientific societies, scientific institutions (i.e. Malbrán Institute) and international agencies (i.e. International Agency for Research on Cancer – IARC). The SAC regularly met to discuss process results and strategies to overcome identified obstacles. Important consideration was given to inclusion of the cytology/pathology societies, as some concern was raised about opposition to the project by the health professionals in charge of reading Pap-smears.

#### Regulation

National guidelines were elaborated and updated through several steps: review of scientific evidence, consultation with national and international experts and approval by the SAC [14]. A provincial ‘resolution’ was enacted with the new protocol, establishing HPV testing as the primary screening method in all public health centres.

#### Policy guidance

National/provincial health authorities signed an agreement regarding a goal for annual screening increases, established as a public health priority for the province. At the provincial level, CHWs were monitored and evaluated in relation to how many of the women they usually visited were tested for HPV. CHW performance was regularly reported to the provincial Health Minister, who had regular meetings with PHC authorities to monitor target achievements.

#### Accountability

The JDP regularly provided provincial health authorities with feedback about project progress and several opportunities existed to monitor process indicators, discuss problems and possible solutions. A list with the main problems identified during project implementation and proposed solutions is presented in Table 3.

The intermediate results of the JDP were presented in national and international scientific meetings and conferences.

### Organisational capacity

#### Service delivery and health workforce

The existing offer of human resources in the public health system was evaluated and considered adequate. Services involved in the screening/diagnosis/treatment process were reorganised; the main axes of this reorganisation were intended to make improvements in the screening process that had been identified as inefficient components of the previous cytology-based screening [12] : decentralisation of cytology reading in small laboratories without quality control, lack of adherence to age and screening frequency recommendations, an informal network for sample transportation and sending of results and use of colposcopy as a screening method. A number of changes were made.

The need of a central cytology/biopsy/HPV laboratory implied centralisation of cytology laboratories from three to one, and reassignment of human resources to the new central laboratory. A central cytology-histology-HPV laboratory was created at the Pablo Soria Hospital, under the supervision of a pathologist. The purpose of this centralisation was to assure high quality laboratory processes and facilitate quality control procedures and coordination between cytology, histology and HPV areas of the health services. In addition, centralising laboratory processes under the supervision of a pathologist was a strategic decision by the NPCCP to limit potential opposition of cytologists to HPV test introduction in Argentina.

Also, a decision was made by the Chief of the Laboratory to not process HPV samples that did not comply with the established age range, frequency and follow-up algorithm. As a result of this strategy, over-screening was 0% and the proportion of screened women at recommended age range was 98.8% (Table 1). Women were informed through the person who took their sample the reason why their test was not analysed; they were reminded of the protocol and date of next HPV testing.

An HPV sample can go 14 days without refrigeration; this characteristic facilitated the reorganisation of sample transport to the laboratory, establishing responsibilities at health centres for assuring transport on specified days. Samples arriving later than 14 days were rejected by the laboratory. Also, the system to send screening results to the health centres was reorganised: It was decided that printed results would no longer be sent to health centres, and instead they would be downloadable from the National screening information system (SITAM). All these decisions were supported by the NPCCP and provincial health authorities.

Screening was promoted through several strategies. First, educational materials were disseminated and displayed at health institutions. Secondly, a TV/radio campaign was broadcasted on local mass media (first year only), however an evaluation suggested that diffusion in TV/radio channels was insufficient and probably had little impact [26]. Third, CHWs were instructed to invite targeted women to get screened during the regular home visits CHWs carried out, for which they received specific training and were provided with specific materials. Two mobile trailers travelled around the province so as to absorb the screening demand generated by the CHW invitation. Finally, during the programmatic implementation of HPV self-collection carried out in 2014, CHWs were provided with a list of the names of women with public health insurance and no previous screening, so they could make a personalised offer of HPV self-collection to this under-screened, socially vulnerable population [15].

Patient navigation was implemented, funded by the NPCCP: two navigators were in charge of contacting women with HPV/abnormal cytology (atypical squamous cells of undetermined significance +) results who had not continued with diagnosis and treatment. In order to do so, navigators identified non-adherent women through SITAM, contacted them through telephone calls or home visits and provided women with specific support [27] (i.e. helping them to find community child care options if the reason why they did not continue the care process was linked to lack of child care). Finally, they shared this contact with the health providers in charge of follow-up/treatment.

In 2012, Self-Collection Modality Study (known as the EMA study) [7], a randomised-controlled trial to evaluate the effectiveness of HPV self-collection was embedded in the JDP. The trial was also a collaborative project between the national/provincial ministries and demonstrated four times more screening among women who had the option to perform self-collection. Based on results of the EMA Project, programmatic HPV self-collection was introduced in 2014 [15] for under-screened women with public health insurance.

The referral network for triage/diagnosis/treatment was reorganised, after careful analysis of the capacity of each unit to respond to the estimated demand. In the case of colposcopy, calculation of that estimation was done considering that it would no longer be used as screening but as a diagnostic method of women with HPV-positive/abnormal Pap results. Annual meetings with colposcopists from the diagnostic network were carried out. These meetings served as refresher courses on colposcopy diagnosis and were also an opportunity to identify problems and obstacles to diagnosis. A key dimension of the training provided to colposcopists was reinforcing the importance of using colposcopy as a tool for diagnosis (rather than screening). To increase women’s access to follow-up, a diagnosis unit was included in the province’s mobile trailers. In total, 18 diagnostic centres and five treatment units provided services to women who need them [24].

Training was provided to health providers from all involved services, i.e. sample takers, colposcopists, laboratory personnel and data entry clerks.

#### Information

Existence of a SITAM guaranteed availability of timely data about monitoring indicators throughout the whole project. SITAM also provided data input to inform decision makers about project evolution, to identify problems and to discuss solutions. A specific HPV module was added to SITAM [28], an easy-to-use tool that displayed monitoring indicators at provincial, hospital and health centre level.

### Health care financing

Funding of HPV testing, training, communication materials, organisation of dissemination events, SITAM maintenance and data entry clerks was provided by the NPCCP. The provincial Ministry of Health funded staff working at the provincial programme, the mobile units, local health providers, diagnostic and treatment infrastructure and the system for sample transport and delivery of results. Services were provided free of cost to women.

### Technology/strategy

#### Effectiveness

International evidence on HPV testing performance was a backbone of the project. It was the basis for the national regulation to introduce HPV testing and was disseminated among health authorities and providers through several scientific meetings and seminars with the participation of well-renowned national and international scientists, which was key for increasing local knowledge and understanding about the methods.

There was wide acceptance among national leaders of the scientific/medical community about the effectiveness of HPV testing and the need for changing the paradigm of cervical cancer screening. Several dissemination activities were carried out to transfer this knowledge and understanding of the utility of the HPV test to health providers. This work was facilitated by the participation of international agencies (i.e. IARC, Pan American Health Organization) and well-renowned national institutions as the Malbrán Institute, which houses the Argentinean HPV laboratory that is part of the WHO-HPV network.

#### Acceptability

A key issue for the acceptability of HPV testing was the understanding of the advantages of HPV testing in relation to cytology-based screening. The reflections of several of the key informants reported in the semi-structured interviews shed light on various aspects of the acceptability of this new technology:

‘We know that we are working with a test that is much more sensitive, less specific in regard to lesions, which means that it subsequently needs the Pap to complement it. However, it allows us to categorize as a lower risk population no less than 87% of the population’.

Acceptability was also linked to an idea that a health problem that had remained unchanged in the last decades could finally be solved by using a new technology:

‘A new technique that breaks the 50-year-long paradigm of the Pap; breaks it, to improve and update it (…)’.

‘For us, everything was new too, and when something is new, it motivates, because sometimes to motivate ourselves, to motivate the women, we need to have other tools. It’s important to have something that isn’t the same old procedure they’ve always known. This method has been very motivational for the teams and the women’.

The HPV test being an opportunity to produce improvements in the health system was also highly valued by the interviewees:

‘(…). It’s been very useful, let’s say that the test has multiple purposes. It’s been useful in what it’s supposed to do and also has parallel achievements like quality control, saving time and money, and taking the resources to the population where it is really needed. In other words, it has lots of benefits’.

Results of interviews showed that decision-makers perceived introduction of HPV testing as a way to focus on women’s health through a project that would allow health authorities to produce modifications in how the health system works.

‘One objective of the Ministry of Health this year [2013] has been women’s health, and we have seen that when we focus on a problem, we obtain good results. By focusing on it (cervical cancer prevention) we have changed reality’.

Interviewees also manifested that HPV test introduction was facilitated by provincial regulation that established HPV testing as the primary screening technique, and support and guidance from the NPCCP:

‘A facilitating factor is (the fact that HPV testing and self-collection) was introduced as a provincial norm, so HPV testing can be provided as standard of care.’

‘A very good thing was the close support by the national team, which always reminded us of the North [direction] of this project; because with the amount of work we have managing the health system, this guidance is crucial for us, so that the urgency of the day to day doesn’t make us lose sight of the other important things’.

The possibility of using HPV self-collection was also seen as an opportunity for the health system to produce a huge change in women’s access to cervical cancer prevention:

‘(...with HPV self-collection…) it is extraordinary to be able to reach a population group that rejects gynecological examinations, or has no access to screening services’.

In general, interviewees also manifested their desire for the JDP and self-collection to be continued as a health policy after project termination:

‘We hope that this is a method that will go on and on for a very long time. We hope it doesn’t end up being just a study, but that it will be implemented as a state policy within the guidelines that state policies have for the provincial government: women’s health (…)’.

## Discussion

In this analysis, we identified key components of the JDP involved in the policy and health system scenarios that made possible the effective introduction of HPV testing in Jujuy [11]. Very limited evidence exists regarding the policy implementation context of HPV testing introduction in low-/middle-income settings; therefore, results of this analysis using the HSF can serve as a model for similar settings.

Stewardship refers to the policy environment in which the implementation of a health strategy is made possible, through three main dimensions: formulating health policy, collecting and using intelligence, and regulation [21]. Health systems from middle-/low-income countries are affected by substantial weaknesses in governance processes and structures, which specially affect the control of chronic diseases [29]. In addition, in contemporary health systems, the number of actors and institutions has multiplied, and authorities deal with a highly complex policy process that can only be governed through processes of steering, coordination and goal-setting, and only by developing a wide range of tools and strategies to this end [21, 30]. In the JDP, several strategies were carried out to strengthen stewardship, including involvement of stakeholders in all phases of the project, and enacting of national and provincial norms that ensured that HPV testing was introduced within a strategic policy framework. Similarly, the Cervical Cancer Prevention in El Salvador (CAPE) project carried out in El Salvador to introduce HPV testing found that the commitment of the national government and other stakeholders such as academic and medical institutions was key to success in the test implementation [31]. In Jujuy, political support was built in a stepwise manner, each phase facilitating the next level of support needed to advance the project. For example, obtaining support and the mandate to introduce HPV testing from provincial programme managers during the annual NPCCP International Seminar provided a basis of legitimacy from which to propose the project to the highest national health authorities, involve them and assure needed resources. At the same time, the support and resources provided by high-level national and provincial authorities were key to obtaining the involvement of second-level health authorities and health providers in Jujuy. In addition, the development of national guidelines and protocols with the involvement of well-recognised international experts and institutions such as IARC-WHO strengthened project viability. International partnerships and collaboration have also been key drivers of the implementation of several pilot or demonstration projects to introduce HPV testing carried out in Central America [32–34].

Involvement of stakeholders from the planning stage of programme implementation has been shown to be a main facilitating factor in the implementation of health innovations [35]. In the JDP, several activities were carried out to build consensus with key actors [24]. National and provincial authorities signed formal agreements and established common objectives and goals. Thus, activities were scheduled and resources allocated accordingly. In addition, protocols were elaborated together with the main scientific societies and institutions through a participatory process. Also, implementation of the strategy was carried out in a complex context that required mechanisms to identify problems and solutions to overcome them. Management roundtables and constant communication between national and provincial teams were key components of those mechanisms. Thus, the concepts of policy guidance, regulation and partnership for collaboration were closely related in the implementation of the strategy.

In many low- and middle-income countries, an important portion of health care is financed through private payment by service users [29, 36]. Evidence has also showed that when financial protection is provided, it is often limited to costs related to some components of care while excluding others which are also essential [29]. The JDP was financed using the regular national and provincial budget, with a health system that provides universal access to health care, assuring financial access to the screening, diagnosis and treatment process. The available funding not only influences the capacity to assure needed resources for the health system, but also indicates what is valued by that system [18]. In Argentina, the National Ministry of Health funded both HPV tests and main project components such as training, communication materials, etc., while the provincial government funded human resources and infrastructure; this certainly acted as a strong signal to the medical community regarding the high priority assigned to cervical cancer prevention. Thus, our results highlight the need for government participation in funding as a condition for building stewardship and programme ownership. In the JDP, funding was totally provided by governmental sources, but evidence from El Salvador has shown that this initial public funding can be partial. Thus, in the CAPE Project carried out in that country, although HPV testing was initially provided through donations, and training and technical support was provided by an international NGO [31], the national government funded personnel and infrastructure. Then, as CAPE expanded, the national government took ownership of the programme; at present, the funding needed to purchase HPV testing is provided by the government [37].

An experiential review of HPV testing implementation in several countries found that a serious disconnect between the priority assigned to cervical cancer prevention in the national policy agenda and the operational support was a common barrier to project scale-up and sustainability [38]. In Argentina, the priority assigned to the JDP was backed up with organisational changes and needed resources. Thus, although the JDP was implemented in a setting where health infrastructure and human resources were evaluated as adequate, substantial changes were carried out: diagnostic and treatment services were reorganised in those components deemed inefficient or of poor quality (i.e. laboratory centralisation); two mobile units travelled around the province to increase screening coverage and facilitate access to diagnosis; a navigator programme provided support to women with abnormal triage; and all providers involved in screening, diagnosis and treatment received extensive training. Evidence from other low-/middle-income countries has shown that organisational change is a major determinant of HPV testing effectiveness. In Central America, introduction of HPV testing was preceded by strengthening the triage and treatment capacity, implementation of information systems and extensive training programmes [32, 34].

Monitoring and evaluation in any health programme is conducted to ensure that processes and systems are developed and adhered to in such a way that the deliverables are of good quality and maximise the benefits to the target population [35]. The use of SITAM in the JDP is an example of an evidence-based monitoring and evaluation process as an essential component of cervical cancer prevention programmes. Using a basic set of indicators on screening, diagnosis and treatment allows for essential feedback that enables providers to measure performance and maximise efficiency [39]. The possibility of continuous monitoring using SITAM was key in the work carried out at the management roundtables, as discussions were rooted in the data and indicators produced by the information system. This underscores the link between stewardship and the availability of information systems for monitoring and evaluation, as accountability is dependent on the existence of data to measure the extent of programmatic problems and produce corrective actions [9].

The JDP had high acceptability by stakeholders, health services and providers, as 100% of provincial health centres and providers adopted the strategy [11] and the main scientific societies participated in the process of building algorithms and protocols with no opposition, including the national cytology and pathology societies. Results from the qualitative interviews showed that this high level of adoption was strongly related to the effectiveness of the strategy, which was seen as a positive innovation. These results contrast with evidence from a study carried out in Dominican Republican showing that some health providers were unsure how a change to HPV testing for screening would address gaps in current cervical cancer screening programmes [40]. In the Dominican study, most providers had limited knowledge of HPV testing as a stand-alone screening test and, very importantly, no initiatives to implement HPV testing in a programmatic context have been carried out there. Technology is one of the three drivers of an opportunity window to implement public policies [41]. In the case of the JDP, HPV testing was perceived as a new, effective technology that was an opportunity to improve upon components of an inefficient cytology-based screening that had been in place for more than 50 years, and this idea was surely reinforced by the organisational changes implemented and public resources committed. Acceptability by health decision-makers was also linked to the possibility of showing results regarding a disease that is considered an indicator of poor women’s health and gender inequality.

The fact that HPV test samples can be self-collected by women was also an important component of the acceptability of the strategy. Self-collection was seen by stakeholders as a tool that gave the health system the possibility to reach a population that is usually out of reach and, in this way, produce a real change in the burden of the disease. During the third year of the JDP, self-collection represented almost 40% of total HPV testing [15], and allowed for a significative increase in screening among socially vulnerable women. Self-collection was offered during home visits by 700 CHWs that are part of the Jujuy Primary Health system [15]; thus, full exploitation of the technology was facilitated by the existence of an extensive network of CHWs that routinely visit households for provision of health services.

Our study presents original evidence regarding the policy context of HPV testing implementation in the JDP. Nonetheless, it has several limitations. First, although the blocks included in HSF are considered as a set of inputs that contribute to the desired outcomes of a health system [42], one of the limitations of the framework is that does not easily capture interaction between the blocks [43]. We have tried to overcome this by identifying links between dimensions, as well as their dynamic interactions. Secondly, the HSF focuses on the health sector and therefore this study does not include in its analysis the research and actions undertaken at the community level to incorporate women’s perceptions and practices in the design and implementation of the strategy. Finally, as they work at the Ministry of Health or were involved in the design and implementation of cervical cancer prevention activities in Argentina, all researchers in this study participated in processes linked to the project under investigation. Such experiences allowed them to gather insights into the implementation process, but, at the same time, may have biased the description of narrated activities.

## Conclusion

This analysis has examined the policy context in which the JDP was implemented and system components that were key for the demonstrated effectiveness of the strategy as well as its high level of population reach and adoption. These are: a high level of political support and consensus among stakeholders, the demonstrated effectiveness of the technology and its acceptability by health authorities and providers, governmental funding of tests and diagnosis/treatment services, implementation of an information system for monitoring and evaluation and the reorganisation of the network of screening, diagnosis and treatment services.

## List of abbreviations

CIN2+, Cervical intraepithelial neoplasia grade 2 or worse; CHWs, Community health workers; HPV, Human Papilloma Virus; HSF, Health System Framework; JDP, Jujuy Demonstration Project; NPCCP, National Programme on Cervical Cancer Prevention; PHC, Primary health care; SAC, Scientific Advisory Committee; SITAM, Sistema de Información para el Tamizaje (National Screening Information System).

## Conflicts of interest

The authors declare that they have no competing interests.

## Funding

This paper presents independent research funded by the Argentinean National Cancer Institute and Consejo Nacional de Investigaciones Científicas y Técnicas (CONICET). The views expressed are those of the authors and not necessarily those of the Argentinean National Cancer Institute or CONICET.

## Authors’ contributions

SA originally conceived the study and secured research support. SA was the principal investigator and study coordinator. MP was the investigator in charge of monitoring and evaluation. RL made substantial contributions to the conception, design and analysis of the study. LT made substantial contributions to study design and implementation. All authors were involved in interpretation of data and critical revision of the manuscript.

## Figures and Tables

**Table 1.** JDP, Argentina (2012–2014). Analysis of impact of HPV test implementation.

Indicator	Outcome
% of women aged 30–64 with public health insurance that were screened at least once	75.0^a^
CIN2+ detection rate among women aged 30+ (odds ratio for clinician-collected HPV test compared to cytology)	2.34 (95% CI: 2.01–2.73)
% of women screened with recommended age	98.8
% of over-screening	0.0
% of HPV positive/abnormal cytology women with colposcopy	74.6
% of women with CIN2+ with registered treatment	83.9
Total number of laboratories processing screening tests (women aged 30+)	Six laboratories centralised in one Cytology/Histology/HPV laboratory
% of inadequate primary test samples	0.2

Source: Adapted from Arrossi *et al* [11], The Lancet Global Health

a This percentage includes clinician-collected tests and self-collected tests for the 5-year period 2012–2017

**Table 2.** HSF. Dimension, sub-dimension, categories and source of data.

Dimension (HSF building block adaptation)	Sub-dimension	Categories	Source of data
Stewardship	Policy support	Consensus building, political support and partnerships for collaboration	Participant observation
	Regulation	Existence of policy guidelines/norms	Secondary documents
	Policy guidance	Formulating sector strategies: Defining goals, directions and spending priorities across services	Participant observation/secondary documents
	Accountability	Monitoring health system performance	Participant observation/secondary documents
Organisational capacity	Service delivery and health workforce	Availability of screening services	Secondary documents
		Strategies to increase screening coverage	Participant observation/secondary documents
		Organisation and strengthening of referral network for triage, diagnosis and treatment	Participant observation/secondary documents
		Availability of health workforce and training	Participant observation/secondary documents
	Information	Availability and use of health information system	Health information system
Health care financing	Funding	Availability of sustainable funding	Secondary documents
Technology/strategy	Evidence based effectiveness	Consensus about the value of the technology/strategy	Secondary documents
	Acceptability	Perception about advantages of technology implementation	Semi-structured interviews

**Table 3.** Problems and agreed-upon strategies implemented during JDP. Jujuy, 2012–2014.

Problem	Agreed-upon strategy
Low screening uptake among target population	Self-collection offered by CHWs with a list of priority women
Delays in delivery/distribution of sample collectors	Organisation of a system to distribute collectors in relation to stock availability in each health centre
Insufficient staff to enter data in SITAM at the HPV laboratory	Incorporation of data entry clerks into the HPV laboratory Data entry of test taking at health centres to reduce data entry burden at the HPV laboratory
Difficulties in sending results to health centres	Printing of results at health centres using SITAM Training of staff from health centres to use SITAM
Low adherence to follow up by women HPV+/Cy−	Elaboration of a list of these women to be distributed among PHC supervisors and CHWs. Recommendation to contact these women whenever possible
Low adherence to triage by women with positive HPV self-collected tests	Active search of these women by CHWs
Differences in diagnostic criteria of colposcopy units	Refresher training of colposcopists
Risk of expiration of HPV tests	Activities at community level to promote HPV testing Promotion in local radios Self-collection established as a priority service provision by CHWs (in relation to other health issues) Close monitoring of CHWs with low numbers of self-collected tests

SITAM, Sistema de Información para el Tamizaje (National screening information system)
